# Supplementary material for: Presenilin 2 N141I mutation induces hyperactive immune response through the epigenetic repression of REV-ERBα
Source: Nat Commun. 2022 Apr 13;13:1972. doi: 10.1038/s41467-022-29653-2 (PMC9008044; doi:10.1038/s41467-022-29653-2)
Supplement: Supplementary file 1 — Supplementary Information [file 41467_2022_29653_MOESM1_ESM.pdf]

Supplementary Information

Presenilin 2 N141I mutation induces hyperactive immune response through the epigenetic repression of REV-ERBα

Hyeri Nam[1], Younghwan Lee[1], Boil Kim[1], Ji-Won Lee[1], Seohyeon Hwang[1], Hyun-Kyu An[1], Kyung Min Chung[1], Youngjin Park[1], Jihyun Hong[1], Kyungjin Kim[1], Eun-Kyoung Kim[1,2], Han Kyoung Choe[1] & Seong-Woon Yu[1]*

[1]Department of Brain and Cognitive Sciences, Daegu Gyeongbuk Institute of Science and Technology (DGIST), Daegu, 42988, Republic of Korea; [2]Neurometabolomics Research Center, DGIST, Daegu, 42988, Republic of Korea

* Correspondence to S.-W. Yu (email: yusw@dgist.ac.kr)

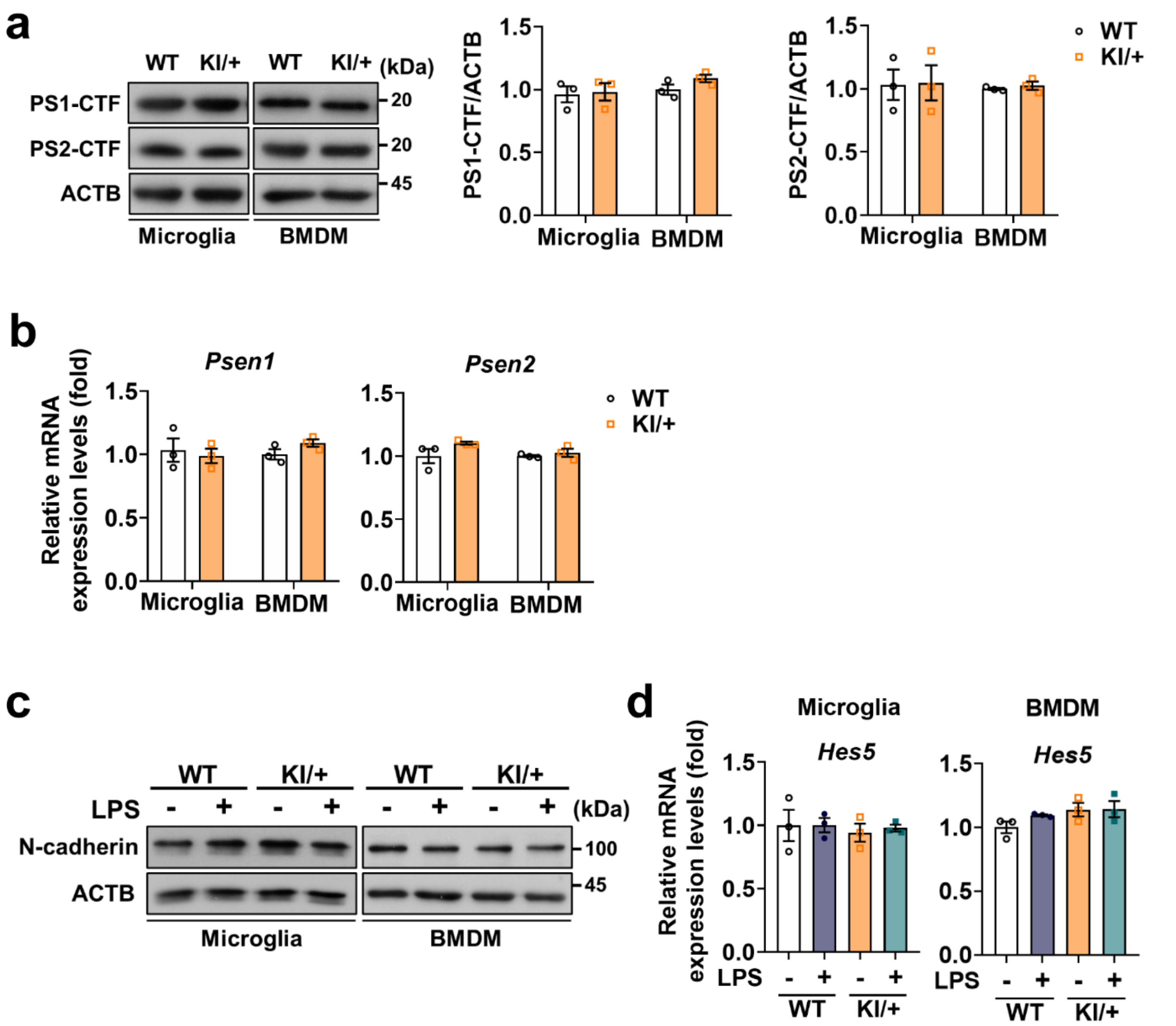

Supplementary Fig. 1 *Psen2* N141I mutation does not change the expression levels of *Psen1* and *Psen2*, or γ-secretase activity in innate immune cells. **a** Western blot analysis of the levels of carboxy-terminal fragments (CTF) of PSEN1 and PSEN2 in primary microglia and BMDM from WT and KI/+ mice. Graphs show quantification of the protein levels (n = 3). **b** Relative mRNA levels of *Psen1* and *Psen2* measured by qRT-PCR in primary microglia and BMDM from WT and KI/+ mice (n = 3). **c**, **d** Representative blots of N-cadherin expression level (**c**; n = 3) and relative mRNA expression levels of *Hes5* (**d**; n = 3) in primary microglia and BMDM from WT and KI/+ mice. mRNA levels were normalized to *Actb*. The blots shown are representative of 3 experiments with similar results. Unpaired t-test for analysis (**a**, **b**) and One-way ANOVA for analysis (**d**). Data are mean ± SEM. Source data are provided as a Source data file.

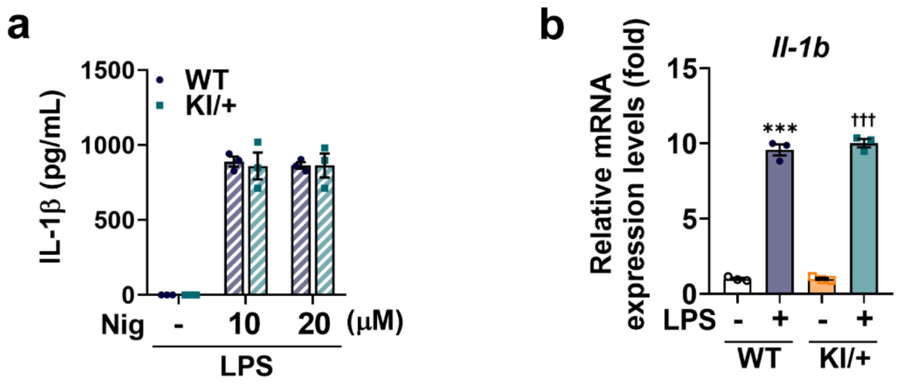

Supplementary Fig. 2 *Psen2* N141I mutation does not affect NLRP3 inflammasome activation and *Il-1b* expression. **a** ELISA of IL-1β in the supernatants from LPS-primed WT and KI/+ microglia after nigericin (10 and 20 μM) treatment (n = 3). Microglia were primed with LPS (1 μg/ mL) for 3 h, followed by nigericin treatment for 40 min. **b** Relative mRNA levels of *Il-1b* in WT and KI/+ microglia after LPS treatment for 12 h. The mRNA levels were normalized to *Actb* (n = 3) (untreated WT vs LPS-treated WT: ***$p < 0.0001$; untreated KI/+ vs LPS-treated KI/+: †††$p < 0.0001$; one-way ANOVA) Data are mean ± SEM. ***$p < 0.001$ vs. untreated WT control. †††$p < 0.001$ vs. untreated KI/+ control. Source data are provided as a Source data file.

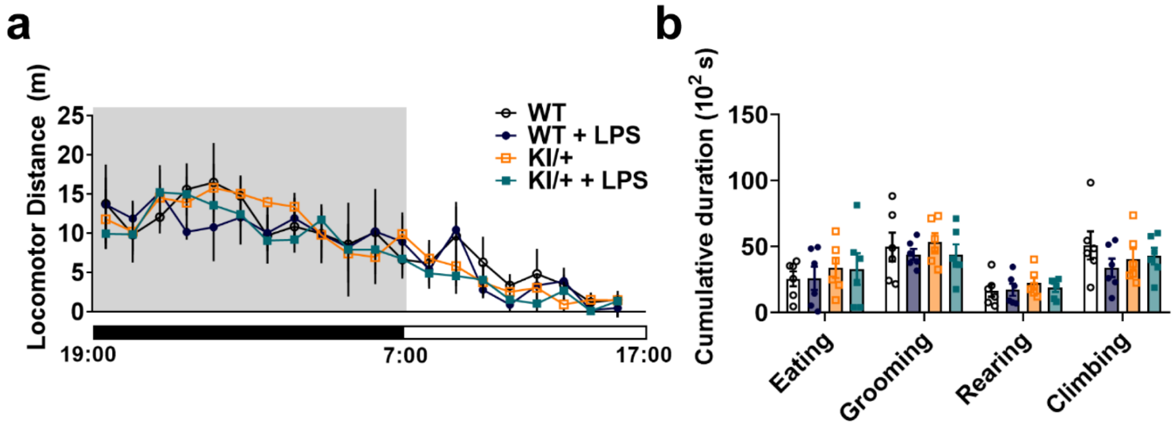

Supplementary Fig. 3 *Psen2*$^{N141I/+}$ mice show normal locomotor activity and behavior in response to LPS. **a**, **b** Analyses of locomotor activity and home cage behavior of WT and KI/+ mice after i.p. injection of LPS (0.35 μg/kg). WT and KI/+ mice were injected with LPS and after 1-h habituation, locomotor activity (**a**; *n* = 3 mice for LPS-injected KI/+; *n* = 4 mice for other group) and general behavior (**b**; eating, grooming, rearing, and climbing; *n* = 6 mice per group) were monitored in LABORAS cages for 22 h. Data are mean ± SEM. Source data are provided as a Source data file.

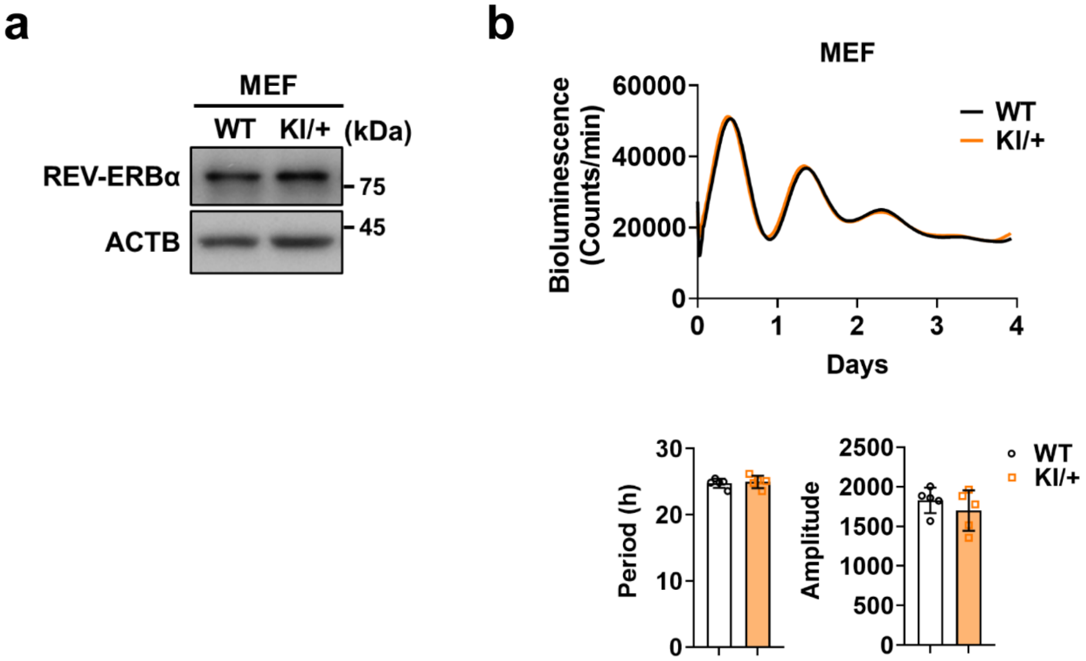

Supplementary Fig. 4 *Psen2* N141I mutation does not affect expression of REV-ERBα and circadian amplitude in mouse embryonic fibroblasts (MEF). **a** Analyses of REV-ERBα protein levels by western blotting in MEF. The blots shown are representative of 3 experiments with similar results. **b** Representative bioluminescence recording of rhythmic *Per2::Luc* expression in MEF obtained from *Per2::Luc*;*Psen2*$^{+/+}$ or *Per2::Luc*;*Psen2*$^{N141I/+}$ mice. Cells were cultured and exposed to DEX (100 nM) for 2 h, followed by measurement of luciferase bioluminescence (each measurement for 1 min with 10-min interval) for 5 days. Circadian period and amplitude were analysed in MEF (n = 5). Unpaired *t-test* for analysis. Data are mean ± SEM. Source data are provided as a Source data file.

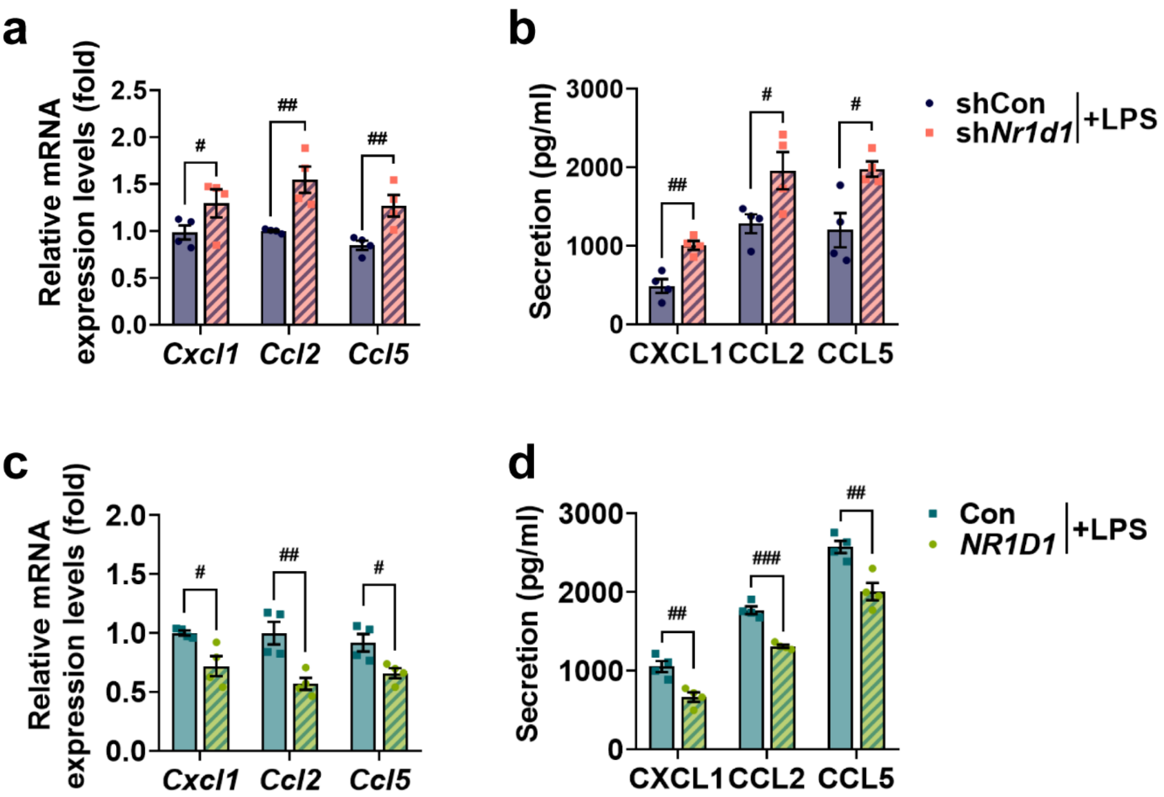

Supplementary Fig. 5 Regulation of clock gene-controlled cytokines is REV-ERBα-dependent. **a, b** mRNA levels (**a**) and secretion (**b**) of CXCL1, CCL2 and CCL5 in WT microglia transduced with sh*Nr1d1* after LPS (1 μg/mL) treatment for 12 h ($n = 4$) (LPS-treated shCon transduced WT vs LPS-treated sh*Nr1d1* transduced WT: #$p = 0.0111$, ##$p = 0.0082$, #$p = 0.0142$, ##$p = 0.0025$, #$p = 0.0438$, and #$p = 0.0174$; unpaired t-test). **c**, **d** mRNA levels (**c**) and secretion (**d**) of CXCL1, CCL2 and CCL5 in *NR1D1*-overexpressing KI/+ microglia after LPS (1 μg/mL) treatment for 12 h ($n = 4$) (LPS-treated Con KI/+ vs LPS-treated *NR1D1* expressed KI/+: #$p = 0.0186$, ##$p = 0.0075$, #$p = 0.0239$, ##$p = 0.006$, ###$p = 0.0001$, and ##$p = 0.0056$; unpaired t-test). mRNA levels were normalized to *Actb*. Data are mean ± SEM. #$p < 0.05$, ##$p < 0.01$, and ###$p < 0.001$ for the indicated comparisons. Source data are provided as a Source data file.

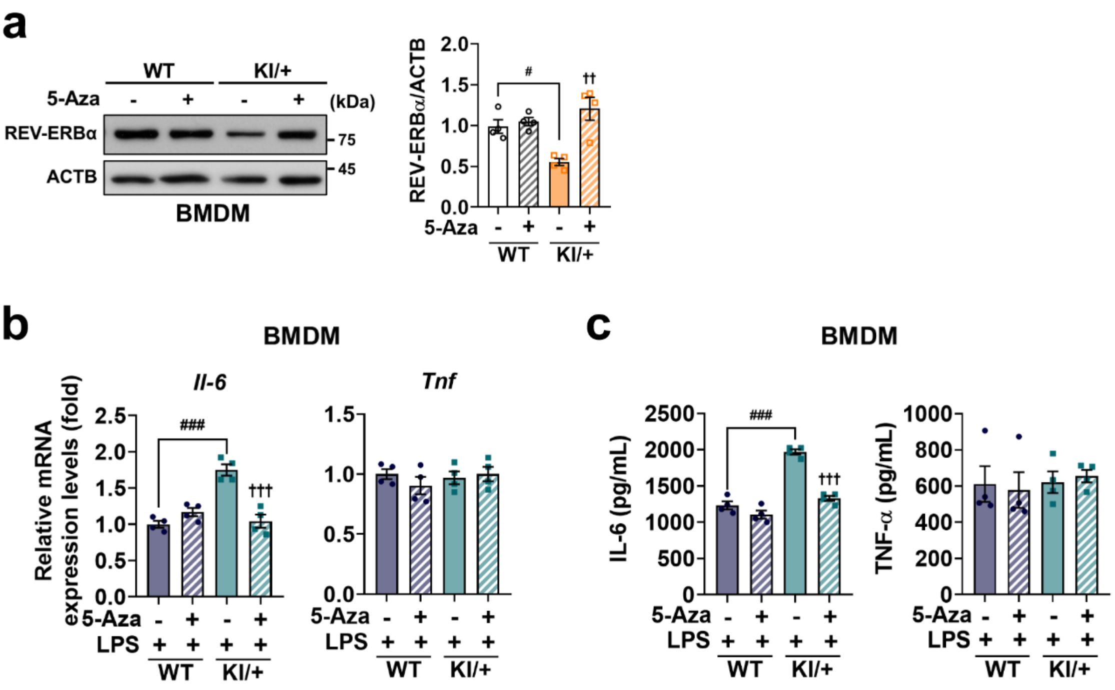

Supplementary Fig. 6 DNA hypermethylation underlies the repression of REV-ERBα in *Psen2* N141I KI/+ BMDM. **a** 5-Azacytidine (5-Aza, 10 μM) treatment for 24 h restores protein levels of REV-ERBα in KI/+ BMDM ($n = 4$) (untreated WT vs untreated KI/+: #$p = 0.0204$; untreated KI/+ vs 5-Aza-treated KI/+: ††$p = 0.0011$; one-way ANOVA). **b**, **c** 5-Aza-induced demethylation reduces the mRNA level (**b**) and secretion (**c**) of IL-6 but not TNF-α in KI/+ BMDM ($n = 4$) (LPS-treated WT vs LPS-treated KI/+: ##<p = 0.0001; LPS-treated KI/+ vs LPS and 5-Aza-treated KI/+: †††p <0.0001; one-way ANOVA). Cells were pre-treated with 5-Aza for 12 h, and mRNA levels and secretion were analysed after co-treatment with LPS for 12 h. mRNA levels were normalized to *Actb*. Data are mean ± SEM. ††$p < 0.01$, and †††$p < 0.001$ vs. untreated KI/+ control. #$p < 0.05$, and ###$p < 0.001$ for the indicated comparisons. Source data are provided as a Source data file.

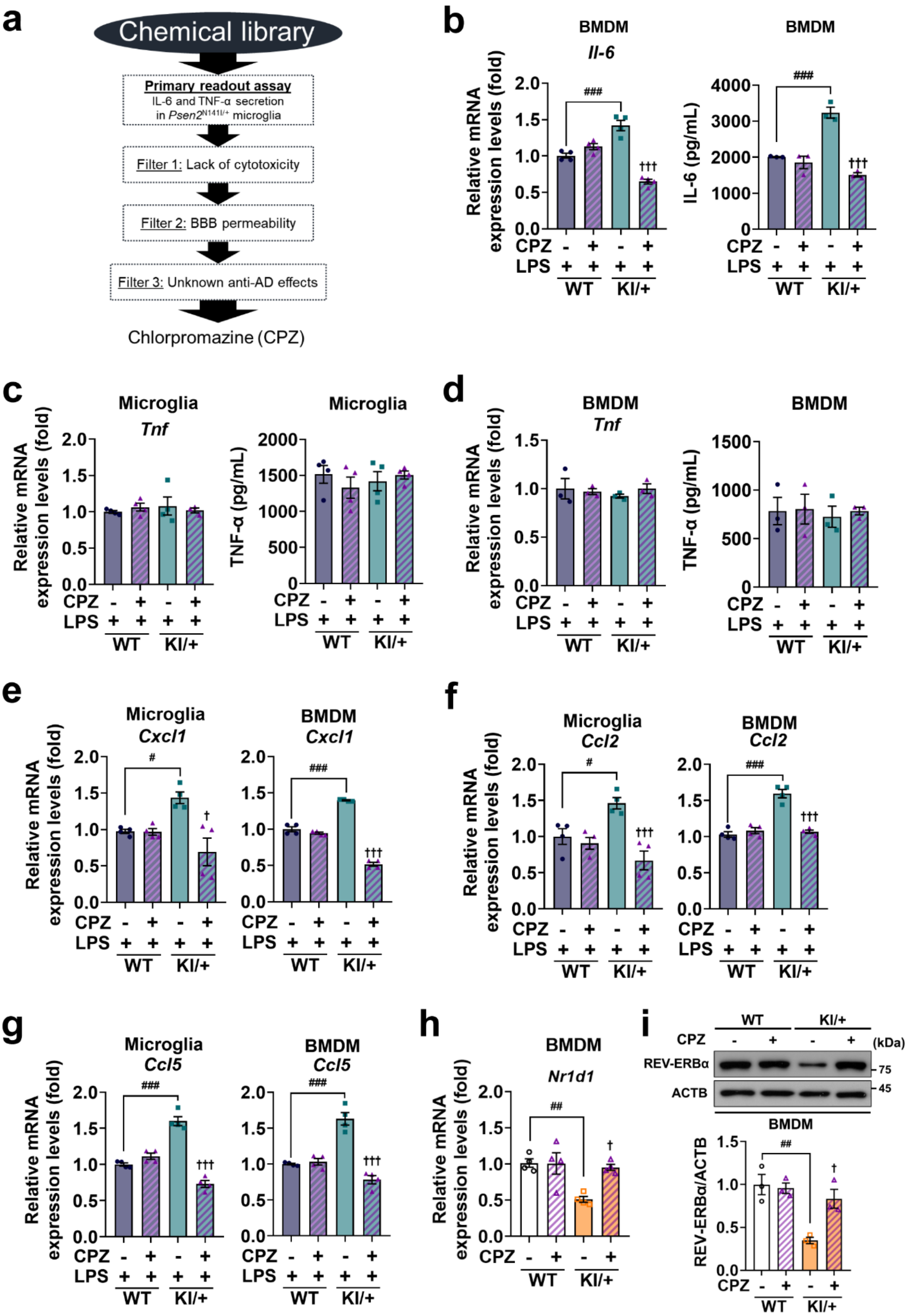

a
Chemical library
Primary readout assay
IL-6 and TNF-α secretion in Psen2N141I/+ microglia
Filter 1: Lack of cytotoxicity
Filter 2: BBB permeability
Filter 3: Unknown anti-AD effects
Chlorpromazine (CPZ)
b
BMDM
Il-6
Relative mRNA expression levels (fold)
IL-6 (pg/mL)
CPZ
LPS
WT
KI/+
c
Microglia
Tnf
TNF-α (pg/mL)
d
BMDM
Tnf
e
Microglia
Cxcl1
BMDM
Cxcl1
f
Microglia
Ccl2
BMDM
Ccl2
g
Microglia
Ccl5
BMDM
Ccl5
h
BMDM
Nr1d1
i
REV-ERBα
ACTB
(kDa)
75
45
REV-ERBα/ACTB

Supplementary Fig. 7 Chlorpromazine selectively reduces expression of clock-controlled cytokines in KI/+ microglia and BMDM. **a** Screening stages. **b** mRNA level (n = 4) and secretion (n = 3) of IL-6 in BMDM (LPS-treated WT vs LPS-treated KI/+: ###p = 0.0002 and ###p = 0.0004; LPS-treated KI/+ vs LPS and CPZ-treated KI/+: †††p < 0.0001; one-way ANOVA). **c**, **d** mRNA level and secretion of TNF-α in microglia (**c**; n = 4) and BMDM (**d**; n = 3). **e-g** mRNA levels of clock-controlled cytokines in microglia and BMDM (n = 4, respectively) (LPS-treated WT vs LPS-treated KI/+: #p = 0.0421, ###p <0.0001, #p = 0.0321, ###p <0.0001, ###p <0.0001, and ###p <0.0001; LPS-treated KI/+ vs LPS and CPZ-treated KI/+: ††p = 0.0016, †††p < 0.0001, †††p = 0.0006, †††p < 0.0001, †††p < 0.0001, and †††p < 0.0001; one-way ANOVA). In all experiments, cells were treated with LPS (1 μg/mL) for 12 h. CPZ (0.5 μM for microglia and 1 μM for BMDM) was treated 30 min prior to LPS. **h**, **i** CPZ treatment restores *Nr1d1* transcript (n = 4) (**h**) and protein (n = 3) levels (**i**) in KI/+ BMDM (untreated WT vs untreated KI/+: ##p = 0.0061 and ##p = 0.0036; untreated KI/+ vs CPZ-treated KI/+: †p = 0.0144 and †p = 0.0192; one-way ANOVA). Data are mean ± SEM. †p < 0.05, and †††p < 0.001 vs. untreated KI/+ control. #p < 0.05, ##p < 0.01, and ###p < 0.001 for the indicated comparisons. Source data are provided as a Source data file.

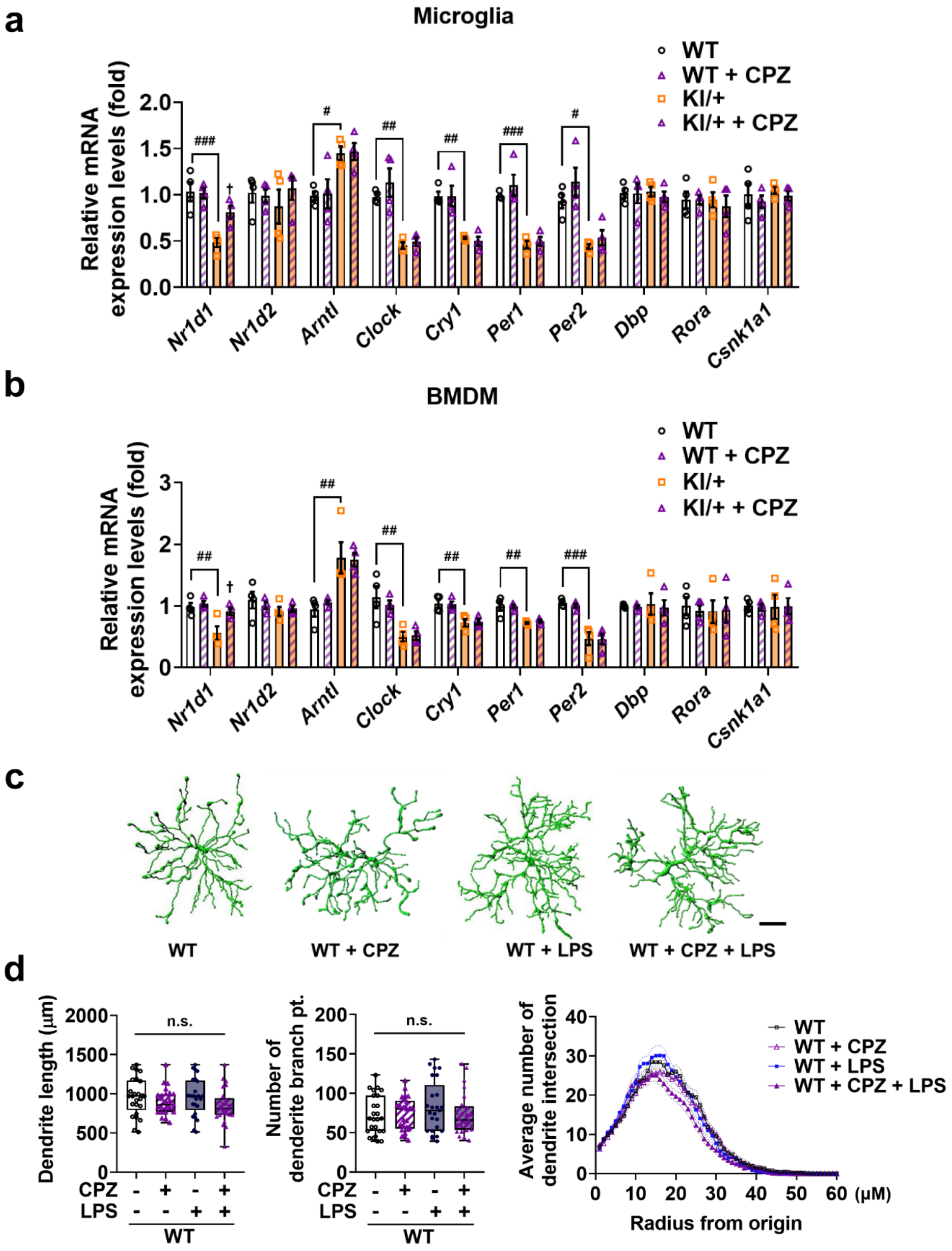
a
Microglia
WT
WT + CPZ
KI/+
KI/+ + CPZ
Relative mRNA expression levels (fold)
2.0
1.5
1.0
0.5
0.0
###
#
##
##
###
#
†
Nr1d1
Nr1d2
Arntl
Clock
Cry1
Per1
Per2
Dbp
Rora
Csnk1a1
b
BMDM
WT
WT + CPZ
KI/+
KI/+ + CPZ
Relative mRNA expression levels (fold)
3
2
1
0
##
##
##
##
##
###
†
Nr1d1
Nr1d2
Arntl
Clock
Cry1
Per1
Per2
Dbp
Rora
Csnk1a1
c
WT
WT + CPZ
WT + LPS
WT + CPZ + LPS
d
Dendrite length (μm)
2000
1500
1000
500
0
n.s.
CPZ - + - +
LPS - - + +
WT
Number of denderite branch pt.
200
150
100
50
0
n.s.
CPZ - + - +
LPS - - + +
WT
Average number of dendrite intersection
40
30
20
10
0
0 10 20 30 40 50 60 (μM)
Radius from origin
WT
WT + CPZ
WT + LPS
WT + CPZ + LPS

Supplementary Fig. 8 Chlorpromazine has no effect on other clock genes expression and microglial morphology in WT mice. **a, b** Comparison of relative mRNA expression levels of clock genes between WT and KI/+ primary microglia and BMDM (n = 4)(untreated WT vs untreated KI/+: ###p = 0.0009, #p = 0.0316, ##p = 0.0034, ##p = 0.0019, ###p = 0.0004, and #p =0.0164 in microglia; ##p = 0.0075, ##p = 0.0085, ##p = 0.0062, ##p = 0.0081, ##p = 0.0028, and ###p = 0.0005 in BMDM; untreated KI/+ vs CPZ-treated KI/+: †p = 0.0343 in microglia; †p = 0.0234 in BMDM; one-way ANOVA). In all experiments, cells were treated with LPS (1 μg/mL) for 12 h. CPZ (0.5 μM for microglia and 1 μM for BMDM) was treated 30 min prior to LPS. **c** Representative 3D filament tracking images of IBA-1 signals made by IMARIS software. Scale bar, 10 μm. **d** Dendrite length, number of branching branch points, and Sholl radius analysis. Data were extracted from FilamentTracker in IMARIS analysis (n = 25 cells from 3 mice per group). One-way ANOVA for analysis. The number of dendrite intersection were analysed by two-way ANOVA. The bounds of the box represent 25th to 75th percentiles ranges. The center lines with the box represent the mean value and whiskers represent the minimum to the maximum range. Data are mean ± SEM. n.s., not significant. †p < 0.05 vs. untreated KI/+ control. #p < 0.05, ##p < 0.01, and ###p < 0.001 for the indicated comparisons. Source data are provided as a Source data file.

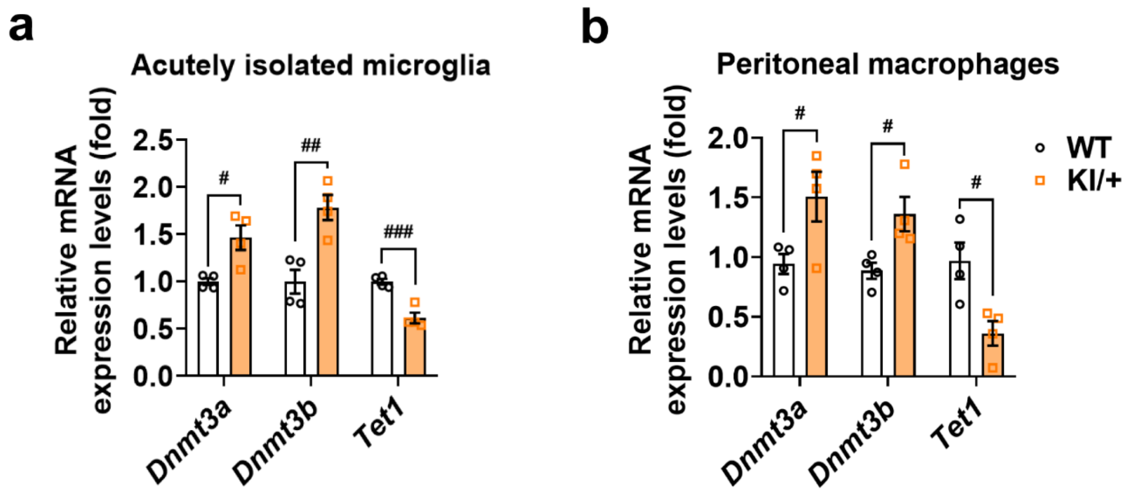

Supplementary Fig. 9 *Psen2* N141I mutation regulates the expression levels of DNA methylases. **a, b** Comparison of relative mRNA expression levels of *Dnmt3a, Dnmt3b, and Tet1* in acutely isolated microglia (**a**; $n = 4$) and peritoneal macrophages (**b**; $n = 4$) between WT and KI/+ mice (8-weeks-old, male) (WT vs KI/+: #$p = 0.0133$, ##$p = 0.0053$, and ###$p = 0.0009$ in acutely isolated microglia; #$p = 0.0456$, #$p = 0.0244$, and #$p = 0.0167$ in peritoneal macrophages; unpaired t-test). mRNA levels were normalized to 18s rRNA. Data are mean ± SEM. #$p < 0.05$, ##$p < 0.01$, and ###$p < 0.001$ for the indicated comparisons. Source data are provided as a Source data file.

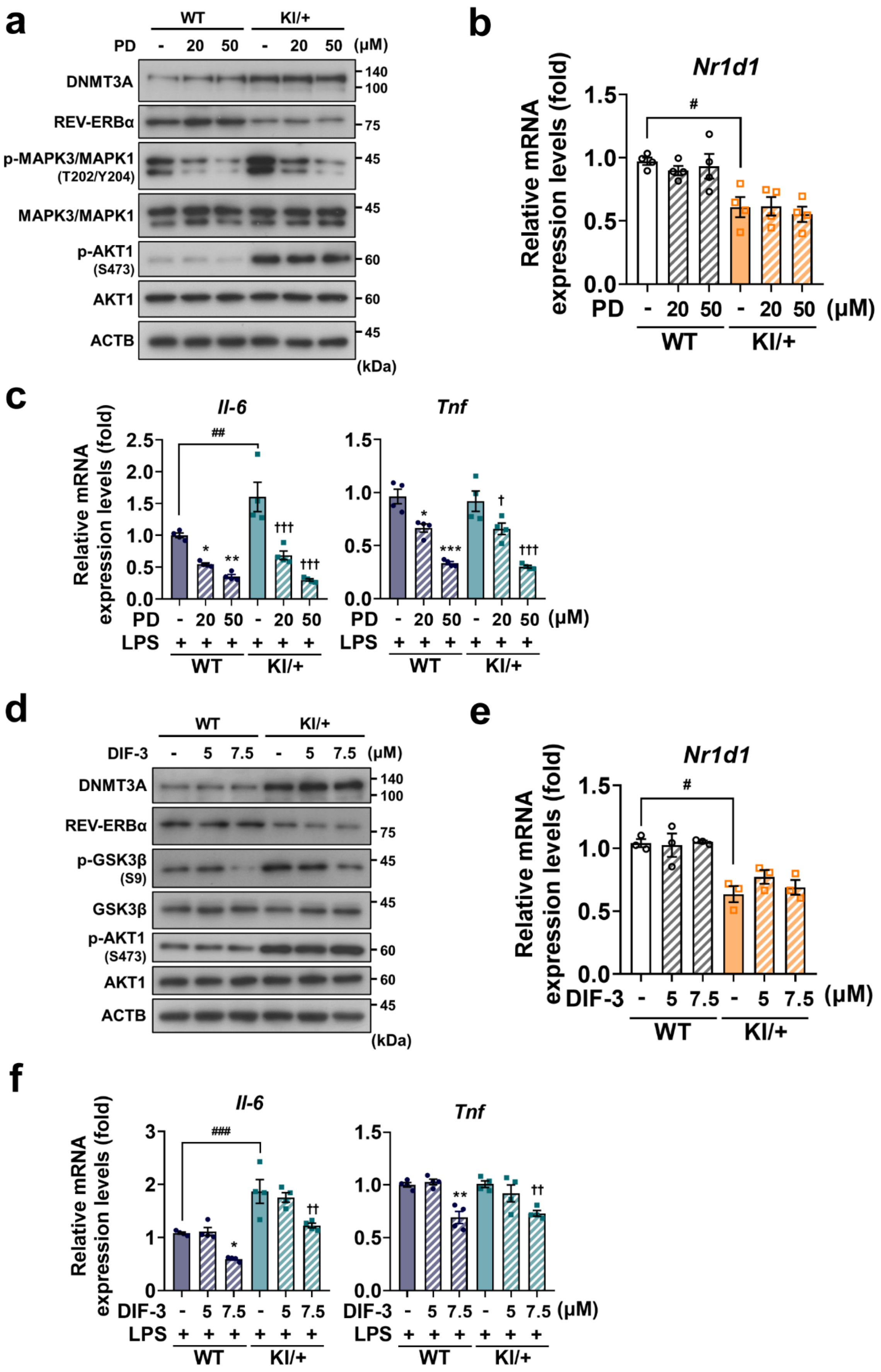
a
WT
KI/+
PD - 20 50 - 20 50 (μM)
DNMT3A
REV-ERBα
p-MAPK3/MAPK1
(T202/Y204)
MAPK3/MAPK1
p-AKT1
(S473)
AKT1
ACTB
140
100
75
45
45
60
60
45
(kDa)
b
Nr1d1
Relative mRNA expression levels (fold)
1.5
1.0
0.5
0.0
#
PD - 20 50 - 20 50 (μM)
WT
KI/+
c
Il-6
Tnf
Relative mRNA expression levels (fold)
2.5
2.0
1.5
1.0
0.5
0.0
##
*
**
†††
†††
1.5
1.0
0.5
0.0
*
***
†
†††
PD - 20 50 - 20 50
PD - 20 50 - 20 50 (μM)
LPS + + + + + +
WT
KI/+
d
WT
KI/+
DIF-3 - 5 7.5 - 5 7.5 (μM)
DNMT3A
REV-ERBα
p-GSK3β
(S9)
GSK3β
p-AKT1
(S473)
AKT1
ACTB
140
100
75
45
45
60
60
45
(kDa)
e
Nr1d1
Relative mRNA expression levels (fold)
1.5
1.0
0.5
0.0
#
DIF-3 - 5 7.5 - 5 7.5 (μM)
WT
KI/+
f
Il-6
Tnf
Relative mRNA expression levels (fold)
3
2
1
0
###
*
††
1.5
1.0
0.5
0.0
**
††
DIF-3 - 5 7.5 - 5 7.5
DIF-3 - 5 7.5 - 5 7.5 (μM)
LPS + + + + + +
WT
KI/+

Supplementary Fig. 10 Inhibition of MAPK1/3 or activation of GSK3β does not rescue *Nr1d1* level in KI/+ microglia. **a** The effects of MAPK1/3 inhibition by PD98059 (PD; 20 and 50 μM) treatment for 12 h on PI3K/AKT activation and expression of DNMT3A and REV-ERBα. **b** *Nr1d1* mRNA expression levels in WT and KI/+ microglia after PD treatment for 12 h ($n = 4$) (untreated WT vs untreated KI/+: #$p = 0.0133$; one-way ANOVA). **c** mRNA levels of *Il-6* and *Tnf* in WT and KI/+ microglia ($n = 4$) (LPS-treated WT vs LPS and PD-treated WT: *$p = 0.05$, **$p = 0.003$, *$p = 0.0149$, and ***$p < 0.0001$; LPS-treated KI/+ vs LPS and PD-treated KI/+: †††$p < 0.0001$, †††$p < 0.0001$, †$p = 0.0381$, and †††$p < 0.0001$; LPS-treated WT vs LPS-treated KI/: ##$p = 0.0057$; one-way ANOVA). Cells were treated with LPS (1 μg/mL) for 12 h. PD was treated 30 min prior to LPS. **d** The effects of GSK3β activation by DIF-3 (5 and 7.5 μM) treatment on PI3K/AKT activation and expression of DNMT3A and REV-ERBα. **e** Relative *Nr1d1* mRNA expression levels in WT and KI/+ microglia after DIF-3 treatment for 3 h ($n = 3$) (untreated WT vs untreated KI/+: #$p = 0.0316$; one-way ANOVA). **f** mRNA levels of *Il-6* and *Tnf* in WT and KI/+ microglia after DIF-3 treatment for 3 h ($n = 4$) (LPS-treated WT vs LPS and PD-treated WT: *$p = 0.0391$ and **$p = 0.0017$; LPS-treated KI/+ vs LPS and PD-treated KI/+: ††$p = 0.0045$ and ††$p = 0.0054$; LPS-treated WT vs LPS-treated KI/: ###$p = 0.0006$; one-way ANOVA). Cells were treated with LPS (1 μg/mL) for 3 h. DIF-3 was treated 30 min prior to LPS. The blots shown are representative of 3 experiments with similar results. mRNA levels were normalized to *Actb*. Data are mean ± SEM. *$p < 0.05$, **$p < 0.01$, and ***$p < 0.001$ vs. untreated WT control. †$p < 0.05$, ††$p < 0.01$, and †††$p < 0.001$ vs. untreated KI/+ control. #$p < 0.05$, ##$p < 0.01$, and ###$p < 0.001$ for the indicated comparisons. Source data are provided as a Source data file.

Table S1. Primers for qRT-PCR, ChIP-qPCR, and methylation-specific PCR analyses

qRT-PCR			
**Gene**	**Gene reference number**	**5′- primer sequence -3′**	
Psen1	NM_001362271	F	CAG TCA CCT GGG GCC TCA TCG
		R	GTT GTG TTC CAG TCT CCA CTG GC
Psen2	NM_011183	F	GTA TGG GGC GAA GCA TGT GAT
		R	ACG CAC AGA CTT GAT AGT GGC
Il-6	NM_031168	F	CTG GAT ATA ATC AGG AAA TTT GC
		R	AAA TCT TTT ACC TCT TGG TTG A
Cxcl1	NM_008176	F	CAT GGC TGG GAT TCA CCT CA
		R	TGA GGT GAA TCC CAG CCA TG
Ccl2	NM_011333	F	GTC CCT GTC ATG CTT CTG GG
		R	CCC CAA GAA GGA ATG GGT CC
Ccl5	NM_013653	F	CCT CAC CAT ATG GCT CGG AC
		R	TGC TCC AAT CTT GCA GTC GT
Tnf	NM_013693	F	CAT CTT CTC AAA ATT CGA GTG ACA A
		R	TGG GAG TAG ACA AGG TAC AAC CC
Hes5	NM_010419	F	GGT ACA GTT CCT GAC CCT GC
		R	GCA GGG TCA GGA ACT GTA CC
Il-1b	NM_008361	F	AGG CCA CAG GTA TTT TGT
		R	GCC CAT CCT CTG TGA CTC
Nr1d1	NM_145434	F	TGC TGG CAT GTC CCA TGA AC
		R	GAC TGT AGG TTG TGC GGC TC
Nr1d2	NM_011584	F	AGC TTC CAG TCC TCG TCC TC
		R	ATC CTG ATG CCA CAT CCC CA
Arntl	NM_007489	F	GCT GTA CCG TCC CTG TCA AA
		R	GGT GCC TTC GTG ACT CTG AA
Clock	AF000998	F	TAG CAC TCC TCC CAG ACA GC
		R	CTA TCA TCC GTG TCC GCT GC
Cry	AF351609	F	CTC GCC TCG GTC CCT TCT AA
		R	CGG AGC TTC TCC CTT GCT TG
Per1	NM_011065	F	CAG TGG AGT CTG GAG GAG GG
		R	GGA GCC AGG AGC TCT GAG AA
Per2	NM_011066	F	GCA GAA ATG CAA AGC AGC CC
		R	GGA TGT TGG CTG GGA ACT CG
Dbp	NM_016974	F	GGA GCC TTC TGC AGG GAA AC
		R	AAA GGC AAA GTG CGT TCC CA
Rora	NM_013646	F	CGC TCG TGG CTT CAG GAA AA
		R	GGA GTC GCA CAA TGT CTG GG
Csnk1a1	NM_146087	F	AGT CGC CGA GAT GAC ATG G
		R	GGG TCC TGA AAA GGA TGC GG
DNMT1	NM_001199431	F	CCG AGA TCC AGA ATG GAG CC
		R	GGC GTC ATA GCC CAT AAG CT
DNMT3a	NM_007872	F	TCA ATG TCA CCC TGG AGC AC
		R	GGG AAC AAC AAC TGC TGC AG
DNMT3b	NM_001003961	F	GGA TAC CAC ACA GGT GGA TGC A
		R	GAG CCA CCA GTT TGT CAG CAG
DNMT3l	NM_019448	F	CCC CGA CTG TAC CAG ATG CTA C
		R	TAT CTC CAT AGG GCC CGC TC

Tet1	NM_001253857	F	CAG AAC AAG TGG GAA GCC ACC
		R	AGG TCC GGG GTA GCA TTC CTA
Tet2	NM_001040400	F	CAG GCC ACA GAG ACT CAA CG
		R	CCG ACA CAA AAG CTT TCT TCC AC
Tet3	NM_001347313	F	CTG AAG GGT GGA TTG TCC CA
		R	GGG AAG TGT GTA CTT GGA GTG G
Actb	NM_007393.5	F	AGA GGG AAA TCG TGC GTG AC
		R	CAA TAG TGA TGA CCT GGC CGT
18s rRNA	X00686	F	GTA ACC CGT TGA ACC CCA TTC
		R	CCA TCC AAT CGG TAG TAG CGA
**ChIP-qPCR**			
**Gene**	**Gene reference number (primer recognition site)**	**5′- primer sequence -3′**	
Il-6	NM_031168 (Promoter region)	F	GGA GAG GGA GTG TGT GTC TT
		R	GTG CTG GTT TAA ATA ACA TCA
Arntl	NM_007489 (Promoter region)	F	GGT TGG AAT TAC AGA CTA CGC C
		R	ACA TTT TAG GAA CAC AGA GTG
Tbp	NM_013684 (Promoter region)	F	CCA CAC CCG CCA CCA GTT CG
		R	TAC AGC CCG GGG AGC ATC GT
Tnf	NM_013693 (Promoter region)	F	ACA CTT CCC AAC TCT CAA GC
		R	TTG TAG AAA GAC CAT GCC TGT
**Methylation-specific PCR**			
**Gene**	**primer type**	**5′- primer sequence -3′**	
Nr1d1 promoter	Methylation	F	GTA AGA CCC TGT CTC AAC ATT CGA GG
		R	TGT GTC AAG TTG ACA CAA AAC TAG CCA G
	Unmethylation	F	ATT GAG TAT GGA TGT TAG T
		R	ACA ATT CAC GAA ATA CAA GAA TA
