# Supplementary material for: Presenilin 2 N141I mutation induces hyperactive immune response through the epigenetic repression of REV-ERBα
Source: Nat Commun. 2022 Apr 13;13:1972. doi: 10.1038/s41467-022-29653-2 (PMC9008044; doi:10.1038/s41467-022-29653-2)
Supplement: Supplementary file 3 — Reporting Summary [file 41467_2022_29653_MOESM3_ESM.pdf]

nature portfolio

Corresponding author(s): Seong-Woon Yu

Last updated by author(s): Feb 9, 2021

# Reporting Summary

Nature Portfolio wishes to improve the reproducibility of the work that we publish. This form provides structure for consistency and transparency in reporting. For further information on Nature Portfolio policies, see our Editorial Policies and the Editorial Policy Checklist.

## Statistics

For all statistical analyses, confirm that the following items are present in the figure legend, table legend, main text, or Methods section.

| n/a | Confirmed | |
|---|---|---|
| ☐ | ☒ | The exact sample size (*n*) for each experimental group/condition, given as a discrete number and unit of measurement |
| ☐ | ☒ | A statement on whether measurements were taken from distinct samples or whether the same sample was measured repeatedly |
| ☐ | ☒ | The statistical test(s) used AND whether they are one- or two-sided<br>*Only common tests should be described solely by name; describe more complex techniques in the Methods section.* |
| ☒ | ☐ | A description of all covariates tested |
| ☒ | ☐ | A description of any assumptions or corrections, such as tests of normality and adjustment for multiple comparisons |
| ☐ | ☒ | A full description of the statistical parameters including central tendency (e.g. means) or other basic estimates (e.g. regression coefficient) AND variation (e.g. standard deviation) or associated estimates of uncertainty (e.g. confidence intervals) |
| ☐ | ☒ | For null hypothesis testing, the test statistic (e.g. *F*, *t*, *r*) with confidence intervals, effect sizes, degrees of freedom and *P* value noted<br>*Give P values as exact values whenever suitable.* |
| ☒ | ☐ | For Bayesian analysis, information on the choice of priors and Markov chain Monte Carlo settings |
| ☒ | ☐ | For hierarchical and complex designs, identification of the appropriate level for tests and full reporting of outcomes |
| ☒ | ☐ | Estimates of effect sizes (e.g. Cohen's *d*, Pearson's *r*), indicating how they were calculated |

*Our web collection on statistics for biologists contains articles on many of the points above.*

## Software and code

Policy information about availability of computer code

| | |
|---|---|
| Data collection | Zeiss ZEN 2010 for Immunofluorescence images; Gen5 2.04 by Synergy HTX for measurement of absorbance, Kronos Dio 2.10.233 by Atto for measurement of bioluminescence; LABORAS 2.0.0 by Metris for measurement of animal behavior |
| Data analysis | Graph Pad Prism 8.0.1 for data analysis; Image studio lite 4.0 by LI-COR Biosciences; for quantification of Western blots; Bio-rad CFX-manager for qRT-PCR; EthoVision XT 11.5 by Nodulus for behavior analysis; IMARIS 9.2.1 by bitplane for 3D image reconstruction and analysis |

For manuscripts utilizing custom algorithms or software that are central to the research but not yet described in published literature, software must be made available to editors and reviewers. We strongly encourage code deposition in a community repository (e.g. GitHub). See the Nature Portfolio guidelines for submitting code & software for further information.

## Data

Policy information about availability of data

All manuscripts must include a data availability statement. This statement should provide the following information, where applicable:

- Accession codes, unique identifiers, or web links for publicly available datasets
- A description of any restrictions on data availability
- For clinical datasets or third party data, please ensure that the statement adheres to our policy

All the data supporting this study are available in the article and Supplementary information. Source data are provided with this paper. The methylation sequencing data generated in this study have been deposited in the Zenodo repository74 and are freely available at: https://doi.org/10.5281/zenodo.5994175.

# Field-specific reporting

Please select the one below that is the best fit for your research. If you are not sure, read the appropriate sections before making your selection.

- [x] Life sciences
- [ ] Behavioural & social sciences
- [ ] Ecological, evolutionary & environmental sciences

For a reference copy of the document with all sections, see nature.com/documents/nr-reporting-summary-flat.pdf

# Life sciences study design

All studies must disclose on these points even when the disclosure is negative.

| | |
|---|---|
| Sample size | Reasonable sample sizes were with the range found in similar methodologies in others' and our former published literatures to certify sufficient reproducibility of the results. The accurate n values used to calculate the statistics are provide in article. |
| Data exclusions | The animals were excluded due to no locomotion in maze or unexpected death (e.g. stereotaxic surgery or anesthesia). The blood serum samples were excluded when there was no reaction on ELISA plate. |
| Replication | All experiments were repeated at least 3 times for calculation of statistical significance and gave similar results and stated in each figure legend. |
| Randomization | Mice with similar age and weight in littermate were randomly and blindly allocated to injection and control experimental groups. For cell culture experiments, samples were randomly chosen for different treatment and control experimental groups and were performed repeatedly. |
| Blinding | Investigators were blinded to group allocation during both data collection (behavior test) and analysis. |

# Reporting for specific materials, systems and methods

We require information from authors about some types of materials, experimental systems and methods used in many studies. Here, indicate whether each material, system or method listed is relevant to your study. If you are not sure if a list item applies to your research, read the appropriate section before selecting a response.

## Materials & experimental systems

| n/a | Involved in the study | |
|---|---|---|
| [ ] | [x] | Antibodies |
| [ ] | [x] | Eukaryotic cell lines |
| [x] | [ ] | Palaeontology and archaeology |
| [ ] | [x] | Animals and other organisms |
| [x] | [ ] | Human research participants |
| [x] | [ ] | Clinical data |
| [x] | [ ] | Dual use research of concern |

## Methods

| n/a | Involved in the study | |
|---|---|---|
| [x] | [ ] | ChIP-seq |
| [x] | [ ] | Flow cytometry |
| [x] | [ ] | MRI-based neuroimaging |

## Antibodies

Antibodies used

The following primary antibodies were used:
Horseradish peroxidase-conjugated β-Actin (Santa Cruz Biotechnology, C-4, sc47778, WB 1:5000)
PSEN1 (Cell signaling, D39D1, 5643, WB 1:1000)
REV-ERBα (Cell signaling, E1Y6D, 13418, WB 1:1000)
DNMT3A (Cell signaling, D23G1, 3598, WB 1:1000)
AKT1 (Cell signaling, C73H10, 2938, WB 1:2000)
p-AKT1 (Ser473, Cell signaling, 9271, WB 1:1000)
p44/42 MAPK (Erk1/2) (Cell signaling, L34F12, 4696, WB 1:1000)
p-p44/42 MAPK (Erk1/2) (Thr202/Tyr204, Cell signaling, D13.14.4E, 4370, WB 1:2000)
GSK-3β (Cell signaling, 27C10, 9315, WB 1:1000)
p-GSK-3β (Ser9, Cell signaling, 9336, WB 1:1000)
N-CADHERIN (BD Biosciences, 32, 610920, WB 1:1000)
PSEN2 (Abcam, EP1515Y, ab51249, WB 1:2000)
Rev-erba (Thermo Fisher Scientific, PA5-29865, WB 1:1000)
Iba-I (FUJIFILM Wako pure chemical corporation, 019-19741, IHC(frozen) 1:250)

Validation

Validation statement of each antibody can be found on the manufacturer's website:
Horseradish peroxidase-conjugated β-Actin (Santa Cruz Biotechnology, C-4, sc47778, https://www.scbt.com/p/beta-actin-antibody-c4?gclid=Cj0KCQiAgP6PBhDmARIsAPWMq6mvKfBhFDrU6DsV-P0zJffiGbUeI8SjOJeVWm7ZYeIKGLVGLUTz8pEaAltWEALw_wcB)

Psen1 (Cell signaling, D39D1, 5643, https://www.cellsignal.com/products/primary-antibodies/presenilin-1-d39d1-rabbit-mab/5643; was validation for WB with Psen1 knock-out cells in laboratory)
Rev-Erbα (Cell signaling, E1Y6D, 13418, https://www.cellsignal.com/products/primary-antibodies/rev-erba-e1y6d-rabbit-mab/13418; was validation for WB with Rev-Erbα knock-out MEF cells in laboratory)
Dnmt3a 3598 (Cell signaling, D23G1, 3598, https://www.cellsignal.com/products/primary-antibodies/dnmt3a-d23g1-rabbit-mab/3598)
AKT1 (Cell signaling, C73H10, 2938, https://www.cellsignal.com/products/primary-antibodies/akt1-c73h10-rabbit-mab/2938)
p-AKT1 (S473, Cell signaling, 9271, https://www.cellsignal.com/products/primary-antibodies/phospho-akt-ser473-antibody/9271)
p44/42 MAPK (Erk1/2) (Cell signaling, L34F12, 4696, https://www.cellsignal.com/products/primary-antibodies/p44-42-mapk-erk1-2-l34f12-mouse-mab/4696)
p-p44/42 MAPK (Erk1/2) (Thr202/Tyr204, Cell signaling, D13.14.4E, 4370, https://www.cellsignal.com/products/primary-antibodies/phospho-p44-42-mapk-erk1-2-thr202-tyr204-d13-14-4e-xp-rabbit-mab/4370)
GSK-3β (Cell signaling, 27C10, 9315, https://www.cellsignal.com/products/primary-antibodies/gsk-3b-27c10-rabbit-mab/9315)
p-GSK-3β (Ser9, Cell signaling, 9336, https://www.cellsignal.com/products/primary-antibodies/phospho-gsk-3b-ser9-antibody/9336)
N-CADHERIN (BD Biosciences, 32, 610920, https://www.bdbiosciences.com/en-us/products/reagents/microscopy-imaging-reagents/immunofluorescence-reagents/purified-mouse-anti-n-cadherin.610920)
PSEN2 (abcam, EP1515Y, ab51249, https://www.abcam.com/presenilin-2ad5-antibody-ep1515y-ab51249.html; was validation for WB with Psen2 knock-out cells in laboratory)
Rev-erba (Thermo Fisher Scientific, PA5-29865, https://www.thermofisher.com/antibody/product/NR1D1-Antibody-Polyclonal/PA5-29865)
Iba-I (FUJIFILM Wako pure chemical corporation, 019-19741, https://labchem-wako.fujifilm.com/us/product/detail/W01W0101-1974.html)

## Eukaryotic cell lines

Policy information about cell lines

| | |
|---|---|
| Cell line source(s) | Lenti-X 293T cells (632180, Clontech) were purchased from Takara. |
| Authentication | Cells were authenticated based on cell morphology and cell size, and no more authentication has been conducted after purchase, because this line does not belong to the list of misidentified cell lines. |
| Mycoplasma contamination | Cell lines were tested negative for mycoplasma contamination. |
| Commonly misidentified lines (See ICLAC register) | N.A. |

## Animals and other organisms

Policy information about studies involving animals; ARRIVE guidelines recommended for reporting animal research

| | |
|---|---|
| Laboratory animals | In this study, 8-weeks-old male Psen2 N141I/+ mice were generated using homologous recombination from C57BL/6J. And 8-weeks-old male Per Per2::Luc;Psen2 N141I/+ mice were used. Animals were maintained in a specific pathogen-free environment under a standard 12-h light/12-h dark cycle at the DGIST animal facility. |
| Wild animals | No wild animals were used in this study |
| Field-collected samples | No filed-collected samples used in this study. |
| Ethics oversight | All protocols and ethical regulations for the care and use of laboratory animals were approved by the Institutional Animal Care and Use Committee of DGIST, Republic of Korea. |

Note that full information on the approval of the study protocol must also be provided in the manuscript.
